# Could the Geriatric Mobile Team trial (GerMoT) inclusion criteria be a pragmatic approach to identifying older adults who may be a relevant target group for integrated care interventions?

**DOI:** 10.1007/s41999-026-01548-1

**Published:** 2026-07-26

**Authors:** Karol R. Biegus, Anne W. Ekdahl

**Affiliations:** 1 Department of Geriatric Medicine, Helsingborg Hospital, Charlotte Yhléns Gata 10, 251 87 Helsingborg, Sweden; 2 Department of Clinical Sciences, Helsingborg, Faculty of Medicine, Lund University, Lund, Sweden

**Keywords:** Multimorbidity, Baseline characteristics, Randomized controlled trial

## Abstract

**Purpose:**

Patients with the traditional definition of multimorbidity, with 2 or more chronic diseases, are a very large and heterogeneous group who differ considerably depending on which set of disorders they suffer from. This paper describes the baselines characteristics of the GerMoT cohort and investigates if the GerMoT inclusion criteria could be used to identify a subgroup of multimorbid patients who may be considered a relevant target group for integrated care interventions.

**Method:**

Participants were 75 years or older, had 3 or more visits to the Emergency Department in the last 18 months, had 3 or more diseases diagnosed according to different ICD-10 chapters and lived in their own home. GerMoT is a randomized-controlled trial comparing proactive outpatient care based on Comprehensive Geriatric Assessment with usual care. The participant’s living arrangements, frailty, grip strength, cognition, physical activity, activities of daily living, quality of life, and physical performance were recorded at baseline.

**Results:**

450 participants were included with a mean age of 83 years (range 75–100); 54% were female. 54% of the cohort lived alone and 44% needed some home adaptation to manage at home. This group had high levels of multimorbidity with a mean of 7.2 (SD = 2.5) diagnoses in different ICD-10 chapters per patient and suffered from pain (80%) and mobility issues (64%). However, they had low levels of frailty (60% non-frail, 13% severely frail) and 90% were independent or partially independent with basic ADLs.

**Conclusion:**

The GerMoT inclusion criteria could be a pragmatic way to identify a subgroup of multimorbid people with frequent emergency department use who may be considered a relevant target group for integrated care interventions.

**Supplementary Information:**

The online version contains supplementary material available at https://doi.org/10.1007/s41999-026-01548-1.

## Key summary points

**Aim:**

To describe the baselines characteristics of the GerMoT cohort and investigate if the GerMoT inclusion criteria could be used to identify a subgroup of multimorbid patients who may be considered a relevant target group for integrated care interventions.

**Findings:**

This group had high levels of multimorbidity, suffered from pain, anxiety, and mobility issues but had low levels of frailty and were relatively independent.

**Message:**

Using the GerMoT inclusion criteria could be a pragmatic way to identify a subgroup of multimorbid patients who may be considered a relevant target group for integrated care interventions.

**Supplementary Information:**

The online version contains supplementary material available at https://doi.org/10.1007/s41999-026-01548-1.

## Introduction

The number of people with two or more chronic health conditions is rising [1, 2]. As a result, high-quality research on older patients with multimorbidity has become crucial for the future of healthcare. However, the absence of a universally accepted definition of “the multimorbid older patient” has hindered research progress.

The most common definition of multimorbidity—the presence of two or more long-term conditions in an individual—captures a heterogeneous group that differ significantly in prognosis, frailty, care needs, independence, and quality of life [3]. As such, it has limited utility when identifying those who would benefit most from targeted interventions. Moreover, the group is large: it includes 23% of the general population and two-thirds of people over 65 [4]. Another study found that 97% of men and 99% of women over 65 meet this definition [5]. This limits the use of this definition in clinical practice as it is not much more selective than a simple age cut-off.

For some people, multimorbidity is associated with complex medical needs, and they may thus benefit from integrated care that takes account of their multimorbidity [6, 7]. In the UK, the National Institute for Health and Care Excellence (NICE) has published a guideline on multimorbidity [8], defined as the presence of two or more long-term conditions. The guideline recommends focusing on a subgroup of patients with complex needs, who may benefit most from integrated care. These include people who struggle with daily activities, rely on multiple services, have both physical and mental health conditions, are frail, have a history of falls, frequently use emergency or other unplanned care, or take multiple regular medications.

While NICE offers some guidance on which patients might have complex needs, the key question remains: how do we easily identify these patients in the community?

In Sweden and many other countries, caring for older adults with multimorbidity is hindered by poor coordination, limited continuity, and insufficient integration of patient perspectives [9] These challenges are especially evident among those living at home, where fragmented care often involves meeting multiple different physicians and nurses due to poor staffing level. In contrast, nursing homes typically benefit from more stable staffing structures [10]. The Swedish National Board of Health and Welfare [11, 12] recommends focusing on multimorbid adults aged ≥ 75 who have had at least three hospital admissions and three diagnoses within the past year. Although this group represents only 7% of Swedes over 75 (0.7% of the total population), they account for 19% of all secondary‑care costs [13]. They can be readily identified through healthcare databases or electronic medical records, which are increasingly used in different countries.

This group has been studied in the Geriatric Mobile Team trial (GerMoT) [14]. GerMoT is a randomized-controlled trial (RCT) on the effects of outpatient care based on Comprehensive Geriatric Assessment (CGA). CGA is a holistic, multidimensional, interdisciplinary diagnostic and treatment process for multimorbid older patients. It is used to address patient’s medical, psychological and functional needs. CGA includes planning and proactive interventions [15]. Participants were randomized into either an intervention group receiving healthcare according to the principles of CGA in addition to usual community-based healthcare, or a control group with access to usual healthcare only [14]. This paper describes the baseline characteristics of the GerMoT cohort to investigate if the GerMoT inclusion criteria could be used to identify a subgroup of multimorbid patients who could benefit from multidimensional healthcare interventions such as CGA.

## Methods

Baseline data from The GerMoT trial were used. Patients meeting the following inclusion criteria were included in the study: ≥ 75 years of age, ≥ 3 visits to the Emergency Department in the last 18 months, and ≥ 3 diseases diagnosed according to different ICD-10 (International Statistical Classification of Diseases and Related Health Problems, 10th revision) chapters, living in their own home and within the catchment area of Helsingborg Hospital. Participants lived in their own home with or without social care.

The trial inclusion criteria were based on the Swedish National Board of Health and Welfare proposed criteria but were adjusted from “at least 3 times admissions in the last 12 months” to “at least 3 visits to the Emergency Department in the last 18 months.” This was done to increase the number of patients recruited as we struggled to meet the number of participants needed as per the power calculation.

### Ethics approval

Ethical approval for the GerMoT trial was obtained from the Regional Ethical Committee in Southern Sweden (2016/630 and 2017/934).

### Consent to participate

All participants provided written informed consent prior to baseline assessment in the GerMoT trial.

### Setting

The GerMoT recruitment place between October 2016 and June 2018 at the Geriatric Medicine Department of Helsingborg Hospital; a medium-sized hospital in the south of Sweden serving approximately 170,000 inhabitants.

### Data collection

Data collection was done before randomization and by assessors that were not involved in the patients’ care. Patient demographic data and information about previous ICD-10 diagnoses were obtained from the electronic Patient Administrative Register of Care in Region Skane [16], an administrative database run by Region Skåne, the county council of the southernmost region of Sweden, that registers healthcare usage including emergency department (ED) and other outpatient visits, admissions, diagnoses, and demographic data. Data from the last 4 years before inclusion were used. The time limit was used as it is thought that any older diagnosis is less likely to be contributing to the patients’ current multimorbidity.

Patient lists were obtained from the database based on inclusion criteria. The research nurse excluded individuals who had died, moved out of the area, or entered a nursing or short-stay home. Eligible patients received a letter about the intervention, followed by a phone call. If interested, a home visit was scheduled, during which further details were provided, questions answered, written consent obtained, and baseline data collected. The study protocol [14] and recruitment process have been described in detail elsewhere [17].

Further data were collected during a protocol-guided interview (Appendix 1) performed by a registered nurse trained in using all the assessment tools. The assessment tool used for measuring frailty was the 9 level Clinical Frailty Scale (CFS), version 1.2 [18], translated into Swedish by Niklas Ekerstad. CFS is a validated screening tool for frailty that focuses on independence, mobility, balance, and cognition. Grip strength was measured using Jamar®dynamometer from Patterson Medical. The best of two attempts was recorded, measured in the dominant hand. Cognition was assessed using the Swedish version [19] of the Montreal Cognitive Assessment (MOCA) tool [20]. MOCA is a screening test of memory, orientation, concentration, attention, and visuospatial and executive abilities. Physical activity was recorded using the modified, short, 4 item version of the International Physical Activity Questionnaire (IPAQ) translated into Swedish [21]. IPAQ 4 item version includes time spent sitting, walking, in moderate and vigorous activity. This is used for self-reported physical activity and participants answered these questions with minimal support from the research nurse. Activities of daily living (ADL) were recorded using Katz Extended ADL Index [22], which includes 6 personal and 4 instrumental ADL measurements. Personal ADL include bathing, feeding, dressing, toilet visits, continence and moving. Instrumental ADL include cleaning, shopping, transport, and cooking. Quality of life (QOL) was assessed using the Swedish version [23] of the 2009 EuroQol Group five-dimension scale (EQ-5D-5L) [24], standard value sets, as produced by the EuroQol Group were used. The domains are mobility, self-care, usual activities, pain/discomfort, and anxiety/depression. Swedish value sets were used [25]. Physical performance was recorded using the Short Physical Performance Battery (SPPB) [26] translated into Swedish [27]. This is used to assess lower extremity function and includes a balance test, a gait speed test, and a chair stand test.

## Results

450 individuals were included in the study with a mean age of 83 years (ranging from 75 to 100), 54% were female. 54% of the cohort lived alone, and 44% need some home adaptation to manage at home. 438 out of the 450 had an MOCA score recorded. The mean score was 22.2 (SD ± 5.74). These results are summarized in Table 1.

**Table 1:** Demographics

Variable	Result
Sex (*n* = 450)	
Female	245 (54%)
Age [years] *n* = 450	
Male, mean (range)	82.5 (75–100)
Female, mean (range)	82.6 (75–99)
Height [cm] *n* = 430	
Male (range)	176 (160–197)
Female (range)	161 (146–181)
Weight [kg] *n* = 424	
Male (range)	80.4 (42–130)
Female (range)	68.4 (36–118)
Marital status (*n* = 450)	
Married/living with partner	211 (46.9%)
Widow/widower	148 (32.9%)
Never married	15 (3.3%)
Divorced, not remarried	69 (15.3%)
Partner that lives separately	7 (1.6%)
Accommodation (*n* = 447)	
Rental	184 (41.2%)
Own house	117 (26.2%)
Own apartment	142 (31.8%)
Other	4 (0.9%)
Aids/adaptation in home* (*n* = 450)	
Yes, new adaptation done	140 (31%)
Yes, adaptation already in place	63 (14%)
No	247 (55%)
Living (*n* = 448)	
Alone	243 (54%)
With partner/spouse	201 (45%)
With children or other	4 (1%)
Education (*n* = 446)	
Incomplete primary education	162 (36%)
Complete primary education	84 (19%)
Complete secondary education	29 (7%)
Folk high school/vocational education	82 (18%)
University/higher education with diploma	84 (19%)
Incomplete higher education or other	5 (1%)
MOCA (*n* = 438)	
Mean score ± SD	22.2 ± 5.74

^*^Home adaptations are provided, if needed, by the municipality to improve independence and mobility at home. These are needs specific and examples include a special shower, ramps, automatic doors, or lights

**Fig. 1:**
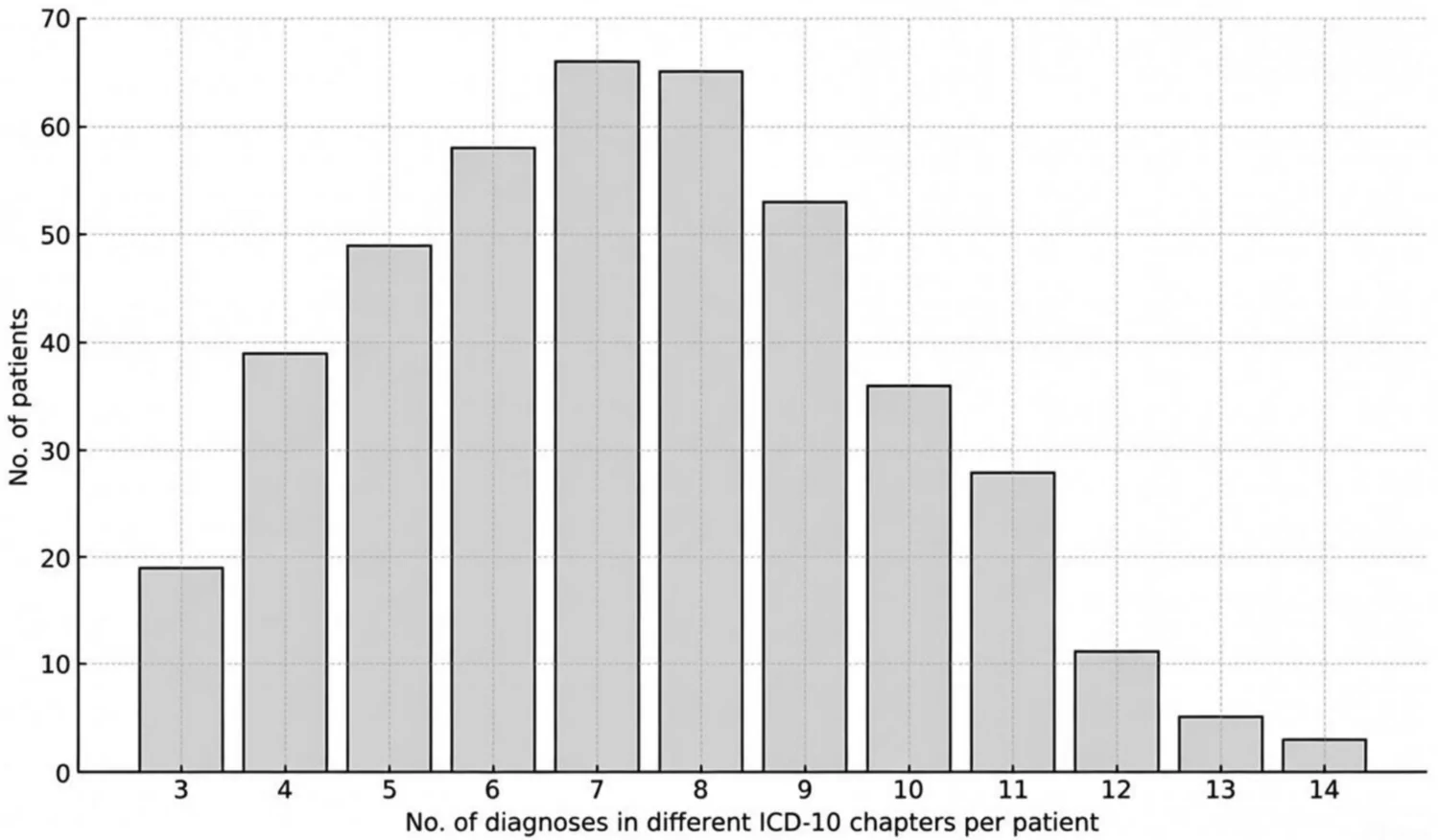
Number of diagnoses. *ICD-10* International Statistical Classification of Diseases and Related Health Problems, 10th revision

Figure 1 shows the number of diagnoses in different ICD-10 chapters participants had.

Table 2 summaries the number of diagnoses the cohort had in each ICD-10 chapter and the number of participants with a diagnosis in each of the chapters. Nearly all participants (96%) had at least one diagnosis in the chapter on the circulatory system.

**Table 2:** Diagnoses divided into ICD-10 chapters

ICD-10 chapters	Number of people with at least one diagnosis in the ICD-10 chapter	Number of separate diagnoses made within each ICD-10 chapter
Certain infectious and parasitic diseases	243 (54%)	474
Neoplasms	234 (52%	466
Diseases of the blood and blood-forming organs and certain disorders involving the immune mechanism	142 (32%)	221
Endocrine, nutritional and metabolic diseases	360 (80%)	889
Mental and behavioral disorders	262 (58%)	509
Diseases of the nervous system	214 (48%)	313
Diseases of the eye and adnexa	331 (74%)	929
Disease of the ear and mastoid process	222 (49%)	371
Diseases of the circulatory system	433 (96%)	1987
Diseases of the respiratory system	309 (69%)	814
Diseases of the digestive system	323 (72%)	943
Diseases of the skin and subcutaneous tissue	321 (71%)	798
Diseases of the musculoskeletal system and connective tissue	391 (87%)	1722
Diseases of the genitourinary system	352 (78%)	897

Physical activity according to IPAQ showed that 52% of people performed very strenuous activity during the last seven days (on a mean of 1.9 days, with a mean of 34.5 min/day) and moderately strenuous activity was performed by 22% of people (on a mean of 3.1 days, with a mean of 79.0 min/day). 65% of people reported performing a 10 min walk during the last 7 days (on a mean of 4.3 days, with a mean of 42.8 min/day). 372 people answered that they spent a mean of 522.3 min/day (8.7 h/day) sitting down. SPPB was performed by 448 people with a mean balance score of 2.5, a mean gait score of 2.4, a mean chair raise score of 1.3, and a mean total of score 6.3.

90% of participants were independent or partially independent with basic ADL, such as feeding, continence, moving, toileting, dressing, and bathing, as can be seen in Fig. 2 in Appendix 2.

443 people performed the hand-grip strength assessment with an overall mean of 22.4 kg. Women had a mean grip strength of 17.5 kg and men 28.2 kg. According to CFS, 265 people (60%) were considered non-frail with a score of 1–4 and 174 people (40%) as frail with a score of 5–9. Figure 3 in Appendix 2 shows a breakdown of the scores.

When using the EQ‐5D‐5L value set for Sweden, our population has a mean value of 0.655 and a median of 0.700. Figure 4 in Appendix 2 shows a breakdown of the different domains.

## Discussion

Patients who fulfilled the inclusion criteria and were willing to participate in the GerMoT trial had high levels of multimorbidity and frequent healthcare contacts. This cohort had problems with pain, anxiety, mobility, and poorer cognition. Despite this, the majority lived alone were relatively independent with basic ADLs and not frail according to CFS.

Patients with the traditional definition of multimorbidity, having at least two chronic conditions [3], are known to have higher mortality [28], substantially higher healthcare utilisation and costs [29, 30], reduced functional capacity [30, 31], and worse QOL [32] or self-reported health status [30] as compared to controls without multimorbidity. In GerMoT, we used a stricter definition of multimorbidity as we required diagnoses in at least three different ICD-10 chapters. The selected cohort would therefore all have exceeded the traditional definition of multimorbidity and have the associated risks.

The participants had a mean of 7 diagnoses in different ICD-10 chapters as can be seen in Fig. 1. This may reflect a broad and complex constellation of healthcare problems and suggests that multimorbidity in our cohort extends far beyond 2 long-term conditions. Only 19 patients had the minimum 3 diagnoses in different ICD-10 chapters needed for the inclusion criteria. The majority had diagnoses within the cardiovascular, musculoskeletal, and connective tissue chapters (Table 2).

The age criterion (≥ 75 years) may be considered unnecessarily restrictive and could exclude younger patients aged 65–74 years who have similar care needs. The age threshold can be used to adjust cohort size. We argue that the age limit is necessary to avoid including younger adults with complex needs arising from syndromes, inherited conditions, or trauma-related disabilities. As their needs may differ from those of multimorbid older adults, they may be less likely to benefit from interventions targeting this population.

There is a clear association between the traditional definition of multimorbidity and increased loneliness [33]. In keeping with this, more than half of our cohort lived alone. Living alone is an independent risk factor for ill health and premature death [34]. Although not synonymous with loneliness, it is a strong risk factor for loneliness [35]. Loneliness in old age is associated with functional and cognitive decline, depression [36–38], and higher healthcare utilization [39].

In our cohort, 41% lived in rented accommodation and 73% had not completed any secondary education. This may reflect poor socioeconomic status [40], a known strong predictor or morbidity and mortality [41–43]. Our findings are in line with the previous studies on the association between lower socioeconomic status and traditional definitions of multimorbidity. Sweden provides strong social support to less well-off citizens, and one could expect that access to, and effects of healthcare are independent of income in this country. However, it has previously been shown that the detrimental effects of lower socioeconomic status on health is strong even within richer countries [44]. These findings should be interpreted with caution as the choice of accommodation and education level in Sweden might be different for a similar cohort in other countries.

Our results showed a distribution skewed towards lower levels of frailty, with few patients with advanced frailty and none with terminal illness. This likely reflects the fact that all participants were living in their own homes and therefore were probably robust enough to maintain at least some degree of independent living. Interventions targeting predominately mildly frail patients may be appropriate for this cohort. Low CFS scores (≤ 4) suggest low medium-term (5-years) risk for death (about 40% or less) or institutionalization (about 20% or less) [18].

Our cohort had low grip strength, a mean of 17.5 kg for women and 28.2 kg for men. Normal values for this age group are quoted at around 23 kg for women and 38 kg for men [45]. Low grip strength is associated with all-cause mortality [46, 47] but may also be a predictor of longer hospital length of stay, and poor physical function [48]. The low grip strength in our cohort may be a marker of poor physical function despite relatively low level of frailty. Grip strength is known to not fully correlate with frailty [49].

Many patients reported doing moderate physical activity or walking but very few did strenuous activities and most reported sitting down on average over 8 h/day. There is good evidence that while any physical activity is beneficial for overall health [50], it is only strength and balance building exercise that have proven benefit in decreasing falls and improving functional performance [51, 52]. Recruiting this cohort of patients to a balance and strength building exercise program may offer potential benefits. It must be noted, however, that IPAQ has not been tested for use in people over the age of 69 years and risk overestimation of activity level, underestimation of sedentary time and could have low reliability for individuals with cognitive impairment. Our results should, therefore, be interpreted with caution [53].

Our cohort had much lower SPPB scores than expected for their age [54], a characteristic associated with decreased level of physical functioning, increased risk of falls [55] and in a nondisabled population predict the subsequent onset of disability [56]. A low score also predicts an increased risk of falls, subsequent decline in ADL status, increased risk for rehospitalisation, and mortality [57]. Healthcare use is particularly high in the GerMoT cohort.

The results for each domain show significantly lower reported QOL than published population norms for Sweden and this age group [58]. Our cohort had least problems with self-care and usual activities. The biggest problems were with pain, anxiety, and mobility. That pain was a serious problem and affects quality of life in this population is also known from previous studies [59, 60].

Our cohort had, on average, low MOCA scores and some may have had undiagnosed dementia [61]. This could make it difficult for them to adequately seek healthcare, leading to frequent ED visits and acute admissions [62, 63]. Our results can be compared to an age-matched cohort from the same region of Sweden who had a mean score of 25.3 (SD 2.5) [64].

One interesting characteristic of our cohort was their relatively high level of independence in basic activities of daily living, including feeding, continence, transfers, toileting, and washing self. This may be explained by the requirement that participants lived in their own homes, as a certain degree of independence in these activities is generally necessary to function well at home. In contrast, participants were less independent in instrumental ADLs, such as cleaning, shopping, transportation, and cooking. These needs can often be met through home-based support services, allowing individuals to remain in their own homes rather than move to a nursing home. It must be noted, however, that we recorded self-reported independence which might differ from objective observations.

This study has no exclusion criteria, no upper age limit, and importantly includes patients with cognitive impairment. This gives a more representative sample of this population. However, despite a consent rate of about 50% [17], there is no record of reasons to decline to participate introducing potential selection bias. In addition, this study was conducted in a well-defined population from a single geographic region. Although some findings may be relevant to similar populations elsewhere, their generalisability is limited. Differences between regions and countries in healthcare systems, reasons for and frequency of ED visits, disease coding practices, social care structures, and the availability of nursing homes will influence the applicability of our findings to other settings. Finally, some of the measurements, such as level of independence and physical activity, are self-reported and should therefore be interpreted with caution.

## Conclusion

The GerMoT inclusion criteria might help to identify a subgroup of people living with multimorbidity who may be considered a relevant target group for multidimensional healthcare interventions such as CGA. The GerMoT cohort of patients had high levels of multimorbidity and healthcare use. They had many risks in common with patients meeting the traditional definition of multimorbidity. However, they suffered in particular from pain, anxiety, and mobility issues and lived alone. This cohort’s predominately mild frailty and relative independence could be useful traits when planning interventions or research on this group. CGA is known to be most effective for those with mild-to-moderate frailty. We believe that the GerMoT inclusion criteria could be a pragmatic approach to identify people who could benefit from CGA and other holistic interventions. The inclusions criteria rely solely on database searches. With the wide use of national identification numbers and electronic medical records in many countries, the GerMoT inclusion criteria could be particularly suited for large-scale interventions or research.

## Supplementary Information

Below is the link to the electronic supplementary material.

Supplementary file1 (DOCX 28 kb)

Supplementary file2 (DOCX 177 kb)

## Data Availability

This research is part of a Ph.D. thesis at Lund University and is in deposit at the University’s repository. The datasets supporting the conclusions of this article are available in the encrypted Lund University repository “LUSEC”, under the name “GerMoT studie” and hyperlink to datasets is http://secure.research.lu.se. They are available from the corresponding author on reasonable request.

## Declarations

### Use of generative AI models

This manuscript has been produced with the assistance of the generative AI model ChatGPT 4o. It has been used for proofreading text, correcting syntax, and spelling errors. The authors have processed any generated text and take full responsibility for the content.
