# Supplementary material for: Could the Geriatric Mobile Team trial (GerMoT) inclusion criteria be a pragmatic approach to identifying older adults who may be a relevant target group for integrated care interventions?
Source: Eur Geriatr Med. 2026 Jul 26;17(5):3009–16. doi: 10.1007/s41999-026-01548-1 (PMC13755077; doi:10.1007/s41999-026-01548-1)
Supplement: Supplementary file 1 [file 41999_2026_1548_MOESM1_ESM.docx]

**Appendix 1**

Protocol 1.4

| **Instrument** | **Result** | **Remark** |
| --- | --- | --- |
| Weight |  |  |
| Height |  |  |
| Grip Strength |  |  |
| Total SPPB Score |  |  |
| ADL (write first independent) |  |  |
| MoCA |  |  |
| CFS |  |  |

**CRF Evaluation of Geriatric Hospital-Associated Team (GerSaT) vs. Regular Care**

**BACKGROUND DATA**

**Total interview time:** Example: 04:03 (4 hours and 3 minutes)

**Gender**

- Man
- Woman

**Marital Status**

- Married/Co-habiting
- Widowed, not remarried
- Divorced, not remarried
- Living apart
- Single
- Other:

**How do you live?**

- In a rental apartment
- In a condo
- In a row house, villa or similar
- Other:

**Do you live together with?**

- Spouse/partner
- Children
- Alone
- Other:

**What is your highest level of education?**

- Primary school or lower
- Secondary school/real exam
- High school diploma
- Folk high school/vocational training
- Incomplete university education or college without degree
- University/college with degree or higher
- Other:

**What was your occupation at retirement?**

**Is your home adapted? (according to your needs)**

- No
- Yes
- Adapted upon moving in

**What adaptations have been made?** If yes to the previous question

**General comments:** (e.g., visual impairment, language barrier, paralysis)

**B. FRAILTY**

Height: _______ cm

Weight:

- Have you unintentionally lost weight in the past year? YES, number of kilos: _____________ Weight:________kg
- NO = 0

The following two statements concern how you have felt in the past WEEK:

**Exhaustion**

- In the past week, everything I have undertaken has been an effort 1 = less than 1 day	2 = 1-2 days	3 = 3-4 days	4 = 4-7 days
- In the past week, I have had difficulty getting things done 1 = less than 1 day	2 = 1-2 days	3 = 3-4 days	4 = 4-7 days

**Grip Strength** Measured for the dominant hand on two occasions (if the dominant arm is injured, use the healthy arm instead)

- Dominant arm: = 1
- Non-dominant but healthy arm = 2

Grip strength attempt 1: _______ kg Grip strength attempt 2: _______ kg

X. Best grip strength value: _______ kg (to be recorded in the database later)

**ACTIVITY HABITS**

Think now about all the very strenuous activities you performed over the past 7 days. Very strenuous physical activity includes activities that are perceived as very strenuous and make you breathe much harder than usual. Think only of activities you performed for at least 10 minutes at a time.

**Over the past 7 days, how many of these days did you perform work that is very strenuous such as heavy lifting, heavy construction and gardening, aerobics, running, or cycling at a high pace?** ______ days ☐ No such activity 🡆 Skip to question 1b

1 b. How much time did you spend on average on such a day on very strenuous physical activity? ______ minutes ☐ Don't know

Think now about all the moderately strenuous activities you performed over the past 7 days. Moderately strenuous physical activity includes activities that are perceived as strenuous and make you breathe somewhat harder than usual. Think only of activities you performed for at least 10 minutes at a time.

**Over the past 7 days, how many of these days did you perform work that is moderately strenuous such as cycling, swimming, moderate construction and gardening, or other activities at a moderate pace? Do not include walking.** ______ days ☐ No such activity 🡆 Skip to question 2b

2 b. How much time did you spend on average on such a day on moderately strenuous activity? ______ hours ______ minutes ☐ Don't know

Think now about all the time you have walked over the past 7 days. This includes walking for transportation.

**Over the past 7 days, how many days did you walk for at least 10 minutes at a time?** ______ days ☐ No walks 🡆 Skip to question 3b

3 b. How much time per day did you spend on average on such a day on walks? ______ hours ______ minutes ☐ Don't know

Think now about the time you have spent sitting during a typical day over the past 7 days in connection with work, studies, transportation, at home, and in your leisure time. For example, time at a desk, with friends, or on the couch watching TV.

**Over the past 7 days, how much time have you spent sitting on such a day?** ______ hours per day ______ minutes per day ☐ Don't know

**Mark by checking ONE box in each group below (like this 🗹) which statement best describes your health TODAY.**

**Mobility**

- I have no problems walking about
- I have slight problems walking about
- I have moderate problems walking about
- I have severe problems walking about
- I am unable to walk about

**Self-care**

- I have no problems washing or dressing myself
- I have slight problems washing or dressing myself
- I have moderate problems washing or dressing myself
- I have severe problems washing or dressing myself
- I am unable to wash or dress myself

**Usual Activities (e.g., work, study, household tasks, family, or leisure activities)**

- I have no problems doing my usual activities
- I have slight problems doing my usual activities
- I have moderate problems doing my usual activities
- I have severe problems doing my usual activities
- I am unable to do my usual activities

**Pain/Discomfort**

- I have no pain or discomfort
- I have slight pain or discomfort
- I have moderate pain or discomfort
- I have severe pain or discomfort
- I have extreme pain or discomfort

**Anxiety/Depression**

- I am not anxious or depressed
- I am slightly anxious or depressed
- I am moderately anxious or depressed
- I am severely anxious or depressed
- I am extremely anxious or depressed

Sweden (Swedish) © 2009 EuroQol Group EQ-5D™ is a trade mark of the EuroQol Group

**ADL Status**

**CLEANING**

- Independent
- Partially independent
- Dependent

**SHOPPING**

- Independent
- Partially independent
- Dependent

**TRANSPORT**

- Independent
- Partially independent
- Dependent

**COOKING**

- Independent
- Partially independent
- Dependent

**BATHING**

- Independent
- Partially independent
- Dependent

**DRESSING**

- Independent
- Partially independent
- Dependent

**TOILETING**

- Independent
- Partially independent
- Dependent

**TRANSFERRING**

- Independent
- Partially independent
- Dependent

**CONTINENCE**

- Independent
- Partially independent
- Dependent

**FEEDING**

- Independent
- Partially independent
- Dependent

**Clinical Frailty Scale (Clinical Frailty Scale^i^) – 9 Levels**

Modified version of the Swedish translation according to the Linköping version (Ekerstad et al. 090917)

- **Very Fit** – robust, active, energetic, and motivated; these people exercise regularly and are among the fittest for their age.
- **Fit** – no active disease but less fit than category 1. Often exercise occasionally but not regularly.
- **Less Fit** – well-controlled chronic disease. Do not exercise regularly except for walking.
- **Vulnerable** – not dependent on others for daily help but often restricted by symptoms.
- **Mildly Frail** – visibly slowing and needing help with more demanding daily activities such as heavy housework or banking. Typically, gradually stop shopping, cooking, and going out alone.
- **Moderately Frail** – need help with all outside activities. Have difficulty with stairs and may need help with bathing. May need assistance with dressing.
- **Severely Frail** – completely dependent on others for daily activities. However, they appear stable and not at high risk of dying within the next 6 months.
- **Very Severely Frail** – completely dependent and approaching the end of life. Typically cannot recover even from a minor illness.
- **Terminally Ill** – expected life expectancy less than 6 months, regardless of help needs.

**Degree of frailty for people with cognitive impairments:** Frailty level corresponds to the degree of dementia divided as follows:

- **Mild Dementia** – forgets recent events. Repeats the same questions and stories. Often withdraws from social contacts.
- **Moderate Dementia** – very difficult to remember recent events but still remembers earlier life. Manages activities of daily living with help.
- **Severe Dementia** – needs help with all activities of daily living to manage.

* 1. Canadian Study on Health & Aging, K. Rockwood et al. A global clinical measure of fitness and frailty in elderly people. CMAJ 2005;173:489-495.
