# Supplementary material for: Could the Geriatric Mobile Team trial (GerMoT) inclusion criteria be a pragmatic approach to identifying older adults who may be a relevant target group for integrated care interventions?
Source: Eur Geriatr Med. 2026 Jul 26;17(5):3009–16. doi: 10.1007/s41999-026-01548-1 (PMC13755077; doi:10.1007/s41999-026-01548-1)
Supplement: Supplementary file 2 [file 41999_2026_1548_MOESM2_ESM.docx]

**Appendix 2**

**Fig. 2** Activities of Daily Living (ADL)

**Fig. 3** Clinical Frailty Scale

**Fig. 4** Quality of life measurements (EQ-5D-5L)
